# Effect of solvophobicity on the phase behavior of linear ABC triblock copolymers in selective solvents: a Monte Carlo study

**DOI:** 10.1039/c8ra05283b

**Published:** 2018-07-30

**Authors:** Zhihua Gao, Jie Cui, Yuanyuan Han, Wei Jiang

**Affiliations:** State Key Laboratory of Polymer Physics and Chemistry, Changchun Institute of Applied Chemistry, Chinese Academy of Sciences Changchun 130022 China yyhan@ciac.ac.cn wjiang@ciac.ac.cn +86-431-85262642; University of Chinese Academy of Sciences Beijing 100049 China; Northeast Normal University, School of Physics Changchun 130024 China

## Abstract

The microphase separation behavior of linear ABC triblock copolymers in A-selective solvents are studied using Monte Carlo simulation. The ABC triblock copolymer used in this study has a short solvophilic block A and two long solvophobic blocks B and C. The effects of the solvophobicity difference and the incompatibility between solvophobic blocks B and C on the micelle morphologies formed by linear ABC triblock copolymers are investigated, and phase diagrams as a function of the solvophobicity of blocks B and C are given at different repulsions between blocks B and C, respectively. A series of multicompartment micelles with distinct solvophobic parts is obtained, such as pupa-like multi-layered micelles, hamburger-like micelles and bumpy disks. Remarkably, when the solvophobicity of blocks B is much stronger than that of blocks C, a novel reverse core–shell–corona micelle with solvophilic blocks A located in the center of the micelle is obtained. Moreover, the results indicate that the competition between the effects of the incompatibility and solvophobicity difference between blocks B and C determines the microphase separation structures in the multicompartment micelles. These simulation results elucidate the mechanism of the formation of ABC triblock copolymer nanostructures and provide theoretical guidance for experimental studies.

## Abstract 2

The solvophobicity difference and incompatibility between different solvophobic blocks determine the overall shapes and micro-structures of micelles formed by linear ABC terpolymers.

## Graphical abstract

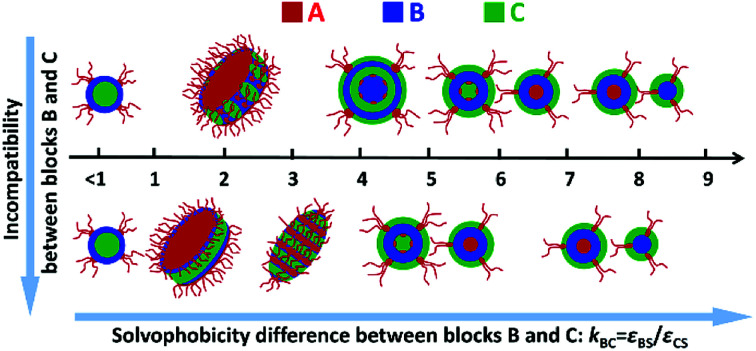

## 1. Introduction

The self-assembly of multiblock copolymers in selective solvents has received great attention due to the formation of micelles with complex nanostructures.^1^ Micelles formed by multiblock copolymers with more than one solvophobic blocks have been found to own multiple subdivided compartments in their solvophobic cores.^2–4^ Micelles with multiple compartments are similar to some biological structures such as eukaryotic cells whose subdivided domains have various physical and chemical properties.^4^ Hence, multicompartment micelles are assumed to be suited for mimicking biological structures and features.^5–7^ Furthermore, more than two incompatible payloads such as gene therapy agents and drug molecules can be encapsulated in their discrete compartments concurrently in a prescribed manner;^8^ therefore, multicompartment micelles also possess potential applications in biomedicine and drug delivery.^8–12^

The design and preparation of multicompartment micelles with precisely controlled microstructures are crucial for the realization of their potential applications. Triblock terpolymers with one soluble block and two different insoluble blocks are thought to be suitable for the preparation of multicompartment micelles because the repulsions between different insoluble blocks can force them to segregate into distinct nanodomains.^13^ The most investigated triblock terpolymer is the ABC miktoarm star terpolymer.^13–19^ For a miktoarm star terpolymer, the length of its solvophilic arm is the key factor to determine its overall micelle morphology, while the length ratio between its two solvophobic arms determines the compartmented structures in the micelles.^19^ By adjusting these two parameters, various multicompartment micelles with delicate microstructures can be obtained.^13–15,19^

In addition to the well-investigated miktoarm star terpolymers, linear ABC triblock copolymers have also attracted significant attention due to their potential in the preparation of multicompartment micelles.^20–30^ For linear ABC triblock copolymers with a solvophilic block A and two sequential solvophobic blocks B and C, core–shell–corona (CSC) spheres are the most commonly formed multicompartment micelles in experiments when the end block C has the strongest solvophobicity.^20–23^ Raspberry-like spheres are another type of multicompartment micelle usually formed by linear ABC triblock copolymers.^21,22,24,25^ Different from the CSC spheres, in the raspberry-like spheres, the middle blocks with a relatively small volume fraction generally aggregate into small bumps rather than a shell on the core. Jiang *et al.* investigated the morphological transition between CSC spheres and raspberry-like spheres using dissipative particle dynamics (DPD) simulation.^26^ They found that the solvophobicity and chain length of the middle block are two key factors in determining the type of spheres. In addition to spherical multicompartment micelles, a variety of cylinders with complex multicompartment structures have also been observed in experiments.^27–30^ For example, the experimental work reported by Cui *et al.* illustrated that the CSC spheres formed by linear ABC triblock copolymers can transform into CSC disks, and even into long cylinders with multiple segmented layers by adjusting the solvent quality.^30^ From these works, we can find that besides the chain length ratio, the solvophobicity of the solvophobic blocks also has significant influence on the micelle structures formed by linear ABC triblock copolymers.

It is evident that the parameter space affecting the self-assembly behaviors of linear ABC triblock copolymers is very large; therefore, simulation methods have become powerful tools for predicting new structures and illustrating the effects of various parameters, such as block solvophobicity,^31–33^ polymer concentration,^34–36^ block length ratio,^32–35^ block sequence^36^ and molecular architecture.^35–37^ Our group has also carried out some simulations on the self-assembly of linear ABC triblock copolymers in selective solvents, *i.e.*, Ma *et al.*^32^ and Zhu *et al.*^33^ illustrated the formation conditions of micelles with bump surfaces using the self-consistent field theory (SCFT) and Monte Carlo (MC) simulation, respectively. In their works, the solvophobicities of the different solvophobic blocks were either quite close or equal. For better understanding the effect of the solvophobicity difference on micelle structures, a wider range of solvophobicities needs to be investigated. Additionally, compared with other factors, investigations on the effect of incompatibility between different solvophobic blocks are still insufficient. Therefore, in this study, the cooperative effect of the solvophobicity difference and incompatibility between different solvophobic blocks on the microphase separation behaviors of linear ABC triblock copolymers is illustrated using the MC method, and phase diagrams as a function of the solvophobicity of blocks B and C with different incompatibility between blocks B and C are obtained.

## 2. Model and method

Lattice Monte Carlo simulations were carried out in a simple cubic box of volume *V* = 50 × 50 × 50. Periodic boundary conditions were employed in three directions of the simulation box. Each lattice site in the simulation box was occupied by either a polymer monomer or a solvent molecule, and the volume fraction of polymers was set as *C*_p_ = 0.08. It should be noted that two monomers cannot occupy one site simultaneously. According to the single-site bond fluctuation model proposed by Carmesin and Kremer^38^ and by Larson,^39,40^ the permitted bond length value adopted by polymer chains is 1 or √2. The microrelaxation model, which has been proven to be highly efficient in relaxing the local chain conformation in the lattice model,^41–43^ was adopted in this study to realize the attempted movements of the monomers in the polymer chains. The microrelaxation model works as follows: a monomer is randomly chosen and we try to exchange it with one of its 18 nearest neighbors. If the chosen neighbor is a solvent molecule, the exchange is accepted if does not violate the bond length restriction. If a single break is created in the chain, the solvent molecule continues to exchange with subsequent monomers along the broken chain until the links reconnect. The exchange is disallowed if it breaks more than two chain connections. The acceptance or rejection of the attempted move is further governed by the Metropolis rule:^44^ if the energy change, Δ*E*, is negative, the exchange is accepted. Otherwise, the exchange is accepted with a probability of *P* = exp[−Δ*E*/(*k*_B_*T*)], where, Δ*E* = Σ_ij_Δ*N*_ij_*ε*_ij_ is the energy change caused by the attempted move; Δ*N*_ij_ is the number difference of the nearest neighbor pairs between components i and j before and after the movement, where, i, j = A, B, C, and S (solvent); *ε*_ij_ is the interaction energy between components i and j; *k*_B_ is the Boltzmann constant and *T* is the temperature. 1/*k*_B_*T* was set as 0.07 in the whole simulation to represent a relatively low temperature.

The linear triblock copolymer studied herein consisted of one short solvophilic block A and two solvophobic blocks B and C, which was denoted as A_2_B_5_C_5_. The chain length (*N*) of the triblock copolymer was unchanged throughout the simulation, *i.e.*, *N* = 12. To mimic the incompatibilities among the different blocks, the repulsive interactions between blocks A and B (or C) were set as *ε*_AB_ = *ε*_AC_ = 0.1, while the repulsive interaction between blocks B and C was set as *ε*_BC_ > 0. Also, to mimic the amphiphilic nature of the ABC triblock copolymers in A-selective solvents, the interaction energy between solvophilic blocks A and the solvents was set as *ε*_AS_ = −0.4, while the interaction energies between the solvophobic blocks B (or C) and solvents were set as *ε*_BS_ (or *ε*_CS_) > 0. To simulate the experimental process when selective solvents are gradually added to a solution, the value of *ε*_BS_ (or *ε*_CS_) gradually increased from 0 to a positive value through 350 steps. At each step, 7000 MC steps (MCS) were carried out (in one MCS, each monomer has to take an attempted exchange move on average). After the value of *ε*_BS_ (or *ε*_CS_) increased to the preset positive value, 200 extra steps with *ε*_BS_ (or *ε*_CS_) unchanged were carried out to confirm the final structures to be in equilibrium state. Besides, all the self-interaction parameters between the same components (*i.e*., *ε*_AA_, *ε*_BB_, *ε*_CC_, and *ε*_SS_) in this study were set as 0. The aforementioned parameter settings ensure that the solvent is good for block A and poor for blocks B and C, and the three blocks were mutually incompatible.

## 3. Results and discussion

In this section, the effect of the solvophobicity difference between blocks B and C on the self-assembly behaviors of linear A_2_B_5_C_5_ triblock copolymers in A-selective solvents were investigated in detail. The values of the interaction parameters *ε*_BS_ and *ε*_CS_ reflect the solvophobicity of blocks B and C, respectively. Different values of *ε*_BS_ and *ε*_CS_ (both ranging from 1.0 to 10.0) were employed for constructing the conditions with various solvophobicity differences. The parameter *k*_BC_ = *ε*_BS_/*ε*_CS_ was introduced to measure the solvophobicity difference between blocks B and C. Since the incompatibility between blocks B and C is another important parameter affecting the micelle morphologies formed by ABC triblock copolymers, two values of the repulsive interactions, *i.e.*, *ε*_BC_ = 2.0 and 4.0, were employed to reflect the weak and strong incompatibilities between block B and C, respectively. It is noteworthy that the two repulsive interactions chosen in this study can lead to either weak (*ε*_BC_ = 2.0) or strong (*ε*_BC_ = 4.0) phase separation between blocks B and C.

### 3.1. Effect of the solvophobicity difference between blocks B and C on the micelle morphologies

**Fig. 1:**
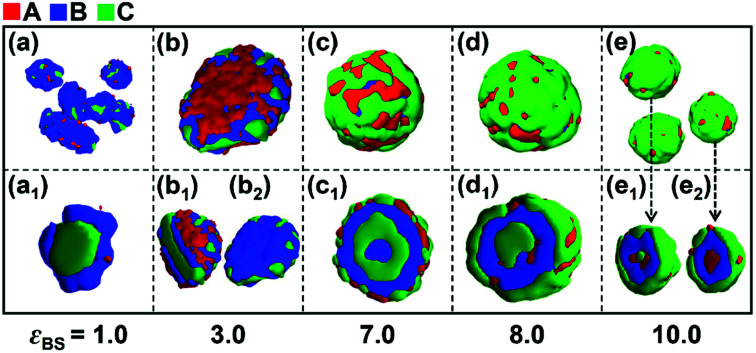
Typical morphologies formed by A_2_B_5_C_5_ triblock copolymers in A-selective solvents as a function of the solvophobicity of blocks B (*ε*_BS_) when the solvophobicity of blocks C and the repulsive interaction between block B and C are *ε*_CS_ = 2.0 and *ε*_BC_ = 2.0, respectively. (a)–(e) are the overall morphologies of the micelles. (a_1_)–(e_1_) and (e_2_) are the cross-sections of the micelles and (b_2_) is the solvophobic parts of the micelle shown in (b).

Firstly, the effect of the solvophobicity difference between blocks B and C on the micelle morphologies were investigated in the case of weak incompatibility between blocks B and C (*i.e.*, *ε*_BC_ = 2.0). Fig. 1 shows the morphological transition of the micelles with different solvophobicities of blocks B (*ε*_BS_) when the solvophobicity of blocks C is *ε*_CS_ = 2.0. As shown in Fig. 1a, when the solvophobicity of blocks B is weaker than that of blocks C (*k*_BC_ = 0.5), typical core–shell–corona (CSC) spheres are observed. The distribution of blocks in these spheres is CBA from the inside to the outside of the micelles (Fig. 1a_1_), which is consistent with the sequence that the most solvophobic blocks C locate in the innermost layer, while the solvophilic blocks A locate in the outermost layer of the micelles. When *ε*_BS_ is increased to 3.0 (*k*_BC_ = 1.5), a disk-like micelle with bumps on its edge is formed (Fig. 1b). In this disk-like micelle, blocks C form bumps on the edge of the disk (Fig. 1b_2_) to reduce the contact between blocks B and solvents, which is mainly because *ε*_CS_ is slightly smaller than *ε*_BS_. When *ε*_BS_ is further increased to 7.0 (*k*_BC_ = 3.5) and 8.0 (*k*_BC_ = 4.0), onion-like spheres with four (Fig. 1c_1_) or three (Fig. 1c_2_) solvophobic layers are formed, respectively. It can be seen from Fig. 1c_1_ and c_2_, blocks B locate in the inner layer, while blocks C and blocks A locate on the surface of the onion-like spheres due to the strong solvophobicity. When the solvophobicity between blocks B and C is extremely large (*ε*_BS_ = 10.0 and *k*_BC_ = 5.0), novel reverse CSC spheres are observed (Fig. 1e_2_). Compared with the normal CSC spheres (Fig. 1a_1_), in the reverse spheres (Fig. 1e_2_), the solvophilic blocks A locate in the innermost layer instead of the surface of the sphere. Xu and coworkers^45^ reported similar reverse micelles formed by amphiphilic ABC triblock copolymers in mixed solvents in experiments. With the change in the solvent polarity, the intramolecular hydrogen bonding among blocks A caused the solvophilic blocks A distributed in the center of the spherical micelles to form reverse micelles. Different from their experimental work, our simulation results indicate that when the solvophobicity of middle blocks B is much stronger than that of the end blocks C, the extremely large solvophobicity difference makes blocks C locate on the surface of the micelles to protect blocks B from contacting with the solvent, and due to the block sequence and the short block length of blocks A, the solvophilic blocks A have to distribute in the center of the micelles.

**Fig. 2:**
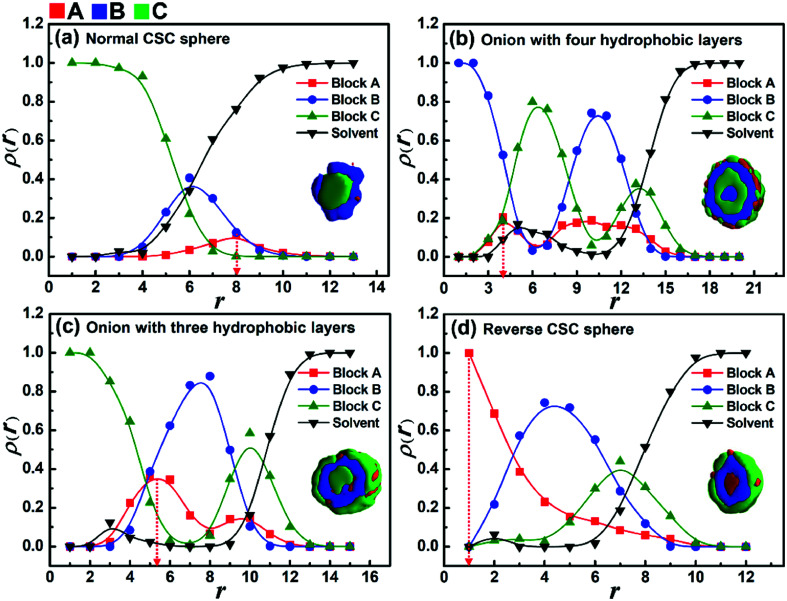
Variations in the densities of components A, B, and C and solvents with the radii (*r*) around the mass center of the spherical micelles with different *ε*_BS_ in the case of *ε*_BC_ = 2.0 and *ε*_CS_ = 2.0. (a) *ε*_BS_ = 1, *k*_BC_ = 0.5; (b) *ε*_BS_ = 7, *k*_BC_ = 3.5; (c) *ε*_BS_ = 8, *k*_BC_ = 4.0; and (d) *ε*_BS_ = 10, *k*_BC_ = 5.0. For clarity, the corresponding cross-section of the typical micelle is given in each figure.

As shown in Fig. 1, when the solvophobicity difference between blocks B and C is increased from *k*_BC_ = 0.5 to 5 in the case of *ε*_CS_ = 2.0, a morphological transition from normal CSC spheres to reverse CSC spheres is observed. During this morphological transition, the translocation of solvophilic blocks A in the micelles apparently plays an important role. Therefore, to better observe the locations of blocks A in each spherical micelle shown in Fig. 1, the variations of the density of block A as well as the other two components with the radii around the mass center (*r*) of the spherical micelles were calculated and shown in Fig. 2. It is noteworthy that for the distribution curve of each block, the position (*r* value) of the highest density value (or peak value) corresponds to the location area of each block. It can be seen from Fig. 2a that blocks C, B and A successively locate from the center (*r* < 4) to the surface (*r* ≈ 8), which is consistent with the morphology of the normal CSC sphere. When the solvophobicity difference is increased to *k*_BC_ = 3.5 (Fig. 2b), two peaks are observed in the density curve of block A, and one of the peak positions (*r* ≈ 4) is quite close to the center of the micelles, indicating that some of the solvophilic blocks A translocated from the surface to the center of the micelle. This phenomenon can also be observed in Fig. 2c. When *k*_BC_ is increased to 5.0 (Fig. 2d), the density curve of block A clearly shows that all of the solvophilic blocks translocated into the center of the micelle, and then reverse CSC spheres are formed. In addition, the density curve of the solvents in the reverse CSC sphere indicates that almost none of the solvents locate in the center of the micelle, which proves that the reverse micelle is a solid sphere rather than a vesicle. The simulation results shown in Fig. 2 indicate that in the case of a small *ε*_CS_, the solvophobicity difference between solvophobic blocks B and C is a key factor in determining the distribution of solvophilic blocks A in the micelles.

**Fig. 3:**
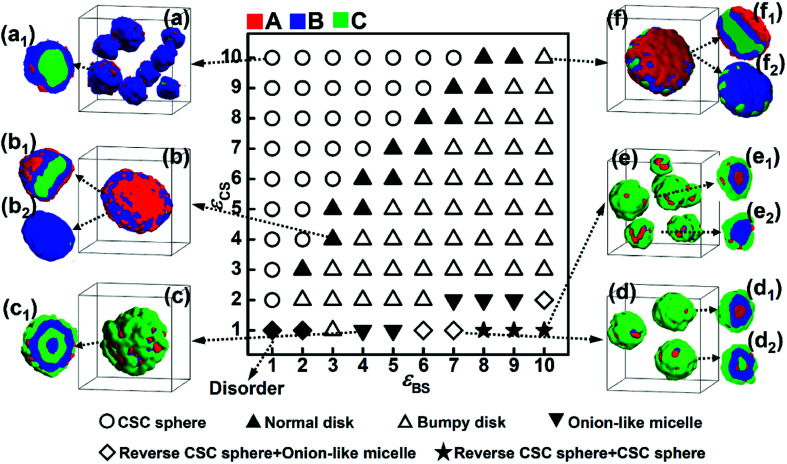
Morphological phase diagram of A_2_B_5_C_5_ triblock copolymers in A-selective solvents as a function of the solvophobicity of blocks B (*ε*_BS_) and C (*ε*_CS_) when the repulsive interaction between blocks B and C is *ε*_BC_ = 2.0. The same symbols in the phase diagram represent the same morphologies, and representative diagrams of the micelle morphologies are given in (a)–(f). (a_1_)–(f_1_), (d_2_) and (e_2_) are the cross-sections of the micelles shown in (a)–(f). (b_2_) and (f_2_) are the morphologies of the solvophobic parts of the micelles shown in (b) and (f), respectively.

According to the simulation results shown in Fig. 1 and 2, increasing the solvophobicity difference between blocks B and C not only affects the overall micelle morphology (Fig. 1), but also the distribution of the solvophilic blocks in the micelles (Fig. 2). Thus, to further understand the effect of the solvophobicity difference on the phase behaviors of ABC triblock copolymers in A-selective solvents, a phase diagram as a function of the solvophobicity of blocks B and C was drawn and shown in Fig. 3. As shown in Fig. 3, when the solvophobicity of blocks B is much weaker than that of blocks C (*ε*_BS_ < *ε*_CS_), normal CSC spheres (Fig. 3a, which are denoted by the circle in the phase diagram) are generally formed. In the normal CSC sphere, the end blocks C form the core of the sphere due to their stronger solvophobicity (Fig. 3a_1_). The formation condition of normal CSC spheres is quite consistent with the experimental reports in the literature.^20–23^ When *ε*_BS_ is increased but still smaller than *ε*_CS_, the disk-like micelles (Fig. 3b, which are denoted by the solid upper triangle in the phase diagram) tend to be formed. Different from the bumpy disk shown in Fig. 1b, in this disk-like micelle, the innermost layer formed by the end blocks C is fully covered by the mid layer formed by blocks B (Fig. 3b_2_). To differentiate this disk-like micelle from the bumpy disk, this type of disk-like micelle is named normal disk in the following discussion. From the relationship between *ε*_BS_ and *ε*_CS_, *i.e.*, *ε*_BS_ < *ε*_CS_, it can be found that the formation of the normal disk is mainly because the solvophobicity of blocks C is stronger than that of blocks B. Therefore, the fully covered middle layer formed by blocks B prevents blocks C from contacting with solvents, which then reduces the free energy of the system. Whereas, when *ε*_BS_ is further increased to meet the condition of *ε*_BS_ ≥ *ε*_CS_, the bumpy disk (Fig. 3f, which is denoted by the open upper triangle) becomes the dominant type of micelle. This is quite reasonable because the bumps formed by blocks C on the edge can increase the contact area between blocks C and the solvents, and also decrease the contact area between blocks B and solvents, which reduces the free energy of the system. In addition to the bumpy disk, in the case of *ε*_BS_ ≥ *ε*_CS_, once the solvophobicity of blocks C is rather weak, *i.e.*, *ε*_CS_ ≤ 2, onion-like micelles (Fig. 3c_1_ and d_2_) and reverse CSC spheres (Fig. 3d_1_ and e_1_) can be found in the phase diagram. In these two micelles, a mixture of solvophobic blocks C and solvophilic blocks A generally forms the outer layers of the micelles, and the solvophobic blocks B are always distributed in the micelles due to their stronger solvophobicity. It should be note that the formation conditions for the onion-like micelles, and especially the reverse CSC spheres, are relatively harsh, *i.e.*, only when the value of *ε*_CS_ is rather small and the solvophobicity difference between *ε*_CS_ and *ε*_BS_ is relatively large, these two kinds of micelles can be formed.

### 3.2. Effect of the incompatibility between blocks B and C on the micelle morphology

**Fig. 4:**
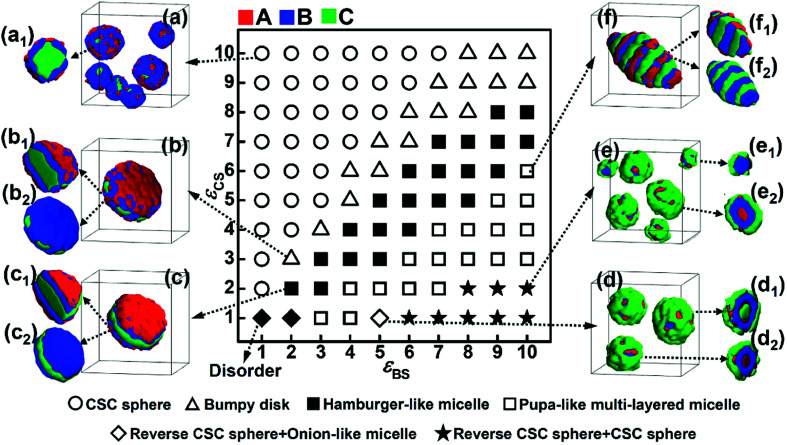
Morphological phase diagram of A_2_B_5_C_5_ triblock copolymers in A-selective solvents as a function of the solvophobicity of blocks B (*ε*_BS_) and C (*ε*_CS_) when the repulsive interaction between blocks B and C is *ε*_BC_ = 4.0. The same symbols in the phase diagram represent the same morphologies, and representative diagrams of the micelle morphologies are given in (a)–(f). (a_1_)–(f_1_), (d_2_) and (e_2_) are the cross-sections of the micelles shown in (a)–(f). (b_2_), (c_2_) and (f_2_) are the morphologies of the solvophobic parts of the micelles shown in (b), (c) and (f), respectively.

The repulsive interaction, *ε*_BC_, reflects the incompatibility between blocks B and C, which is assumed to be an important factor affecting the microphase structures self-assembled by ABC terpolymers.^13^ In this subsection, the repulsive interaction between blocks B and C was increased to *ε*_BC_ = 4.0, and the phase diagram as a function of *ε*_BS_ and *ε*_CS_ was obtained (Fig. 4). The comparison between the two phase diagrams with either weak (Fig. 3) or strong (Fig. 4) repulsive interactions between blocks B and C was done to illustrate the effect of the incompatibility between these two blocks on the self-assembly behaviors of ABC triblock copolymers.

As shown in Fig. 4, when the solvophobicity of blocks C is much larger than that of blocks B, the normal CSC sphere (Fig. 4a), which is denoted by the circle in the top left corner of the phase diagram, is the dominant type of micelle. In contrast, when the solvophobicity of blocks C is much smaller than that of blocks B, the onion like micelle (Fig. 4d_1_) and reverse CSC sphere (Fig. 4d_2_ and e_2_), which are denoted by the diamond and star in the bottom right corner of the phase diagram, respectively, can generally be observed. By comparing Fig. 4 with 3, it can be found that the formation conditions of the aforementioned three micelles in the case of *ε*_BC_ = 4.0 are quite similar with that in the case of *ε*_BC_ = 2.0. This indicates that when the solvophobicity difference between blocks B and C is large, the micelle morphology is independent of the incompatibility between blocks B and C and mainly dependent of the relationship between *ε*_BS_ and *ε*_CS_. Specifically, when *ε*_BS_ is much smaller than *ε*_CS_, the normal CSC sphere is formed, whereas, when *ε*_BS_ is much larger than *ε*_CS_, the onion-like micelle and reverse CSC sphere are formed. However, when the solvophobicity difference between blocks B and C is relatively small (*i.e.*, the values of *ε*_BS_ and *ε*_CS_ are close), the influence of the incompatibility between blocks B and C becomes stronger. It can be seen from Fig. 4 that when the values of *ε*_BS_ and *ε*_CS_ are close, the bumpy disk (Fig. 4b, denoted by the open upper triangle in the phase diagram) generally forms in the case of *ε*_BS_ < *ε*_CS_, while the hamburger-like micelle (Fig. 4c, denoted by the solid square in the phase diagram) generally forms in the case of *ε*_BS_ ≈ *ε*_CS_, and the pupa-like multi-layered micelle (Fig. 4f, denoted by the open solid square in the phase diagram) generally forms in the case of *ε*_BS_ > *ε*_CS_. In other words, morphological transitions from bumpy disk to hamburger-like micelle, and then to pupa-like multi-layered micelle can be observed with an increase *ε*_BS_. This is different from the phase diagram shown in Fig. 3, in which only the normal disk (Fig. 3b) and bumpy disk (Fig. 3f) can be observed when the values of *ε*_BS_ and *ε*_CS_ are close. Therefore, it can be found that when the solvophobicity difference between blocks B and C is relatively small, an increase in the incompatibility between blocks B and C makes the micelle morphology more variable. It is noteworthy that similar pupa-like multi-layered micelles formed by ABC star terpolymers have been observed in the case of an extremely strong incompatibility between components B and C by Kong *et al.* using MC simulation.^19^ Our simulation results show that the pupa-like multi-layered micelle can also be obtained by adjusting the solvophobicities of blocks B and C. In our previous study, we investigated the phase behaviors of poly(2-vinylpyridine)-*b*-polybutadiene-*b*-polystyrene (P2VP-*b*-PBd-*b*-PS) in selective solvents,^32^ where a mixture of toluene and methanol was used as the selective solvent. By adjusting the volume ratio of toluene to methanol, the solvent quality can be easily changed, and the solvophobicity of PS can either be stronger or weaker than that of PBd. The experimental result showed that when the solvophobicity of PS is weaker than that of PBd, the bumpy disk can be formed, which is quite consistent with our simulation results (Fig. 3f). Therefore, it can be concluded that by carefully controlling the solvent quality, other multicompartment micelles, such as the pupa-like multi-layered micelles (Fig. 4f) and reverse CSC spheres (Fig. 4e) may also be obtained from linear ABC triblock copolymers in experiments.

In addition, generally in experiments, strong incompatibility between different insoluble blocks is assumed to be essential in preparing multicompartment micelles.^13^ However, our simulation results illustrate that a relatively weak incompatibility between blocks B and C can still lead to phase separation between the different insoluble blocks (Fig. 3). By adjusting the incompatibility and the solvophobicity difference, a variety of multicompartment micelles with different micro-phase separation structures can be formed (Fig. 3 and 4).

### 3.3. The microphase separation behaviors of solvophobic blocks B and C

To further elucidate the effects of the incompatibility and the solvophobicity difference between blocks B and C on their microphase separation behaviors, the average contact numbers between blocks B and C (*N*_BC_), which reflects the microphase separation degree between blocks B and C, were calculated.

**Fig. 5:**
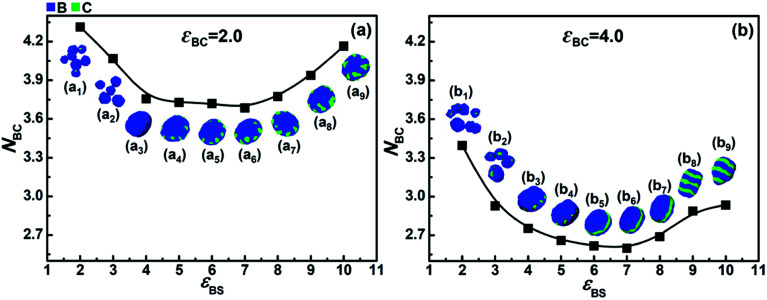
Variations in the average contact numbers between blocks B and C (*N*_BC_) with *ε*_BS_ in the case of (a) *ε*_BC_ = 2.0 and (b) *ε*_BC_ = 4.0, respectively. The solvophobicity of blocks C is *ε*_CS_ = 5.0. (a_1_)–(a_9_) and (b_1_)–(b_9_) are the morphologies of the solvophobic parts of the corresponding micelles.

Fig. 5a shows the variation of *N*_BC_ with *ε*_BS_ when *ε*_CS_ = 5.0 and *ε*_BC_ = 2.0. It can be seen that when the solvophobicity difference between blocks B and C is rather small (*i.e.*, *ε*_BS_ = 5.0–7.0), the value of *N*_BC_ is very low, which indicates that the microphase separation between blocks B and C is sufficient. However, when the solvophobicity difference is large (*i.e.*, *ε*_BS_ < 5.0 and *ε*_BS_ > 7.0), the value of *N*_BC_ is relatively high. A high value of *N*_BC_ means that the microphase separation between blocks B and C is insufficient, and therefore, we can infer that it is the solvophobicity difference rather than the incompatibility between blocks B and C that determines their microphase structure. Taking the case with the largest solvophobicity difference as an example (*i.e.*, *ε*_BS_ = 10.0), it can be seen that a disk-like micelle with large bumps (composed of blocks C) on the edge is formed (Fig. 5a_9_). According to their high *N*_BC_ value, the large C bumps apparently increase the contact area between the incompatible blocks B and C. However, the formation of large C bumps also reduces the contact area between the solvents and blocks B. Hence, the formation of large C bumps mainly results from the solvophobicity difference between blocks B and C. This reveals the fact that there is competition between the effects of the incompatibility and the solvophobicity difference on the microphase structures formed by blocks B and C. When the incompatibility between blocks B and C is much stronger, *i.e.*, *ε*_BC_ = 4.0, as shown in Fig. 5b, all the *N*_BC_ values are much smaller than that in Fig. 5a. This indicates that the microphase separation between blocks B and C is always stronger in the case of *ε*_BC_ = 4.0 than that in the case of *ε*_BC_ = 2.0. However, the overall trend of the *N*_BC_ variation shown in Fig. 5b is quite similar to that shown in Fig. 5a, which indicates that the competition between the incompatibility and the solvophobicity difference between blocks B and C still exists when their incompatibility is strong. It should be noted that more microphase structures (Fig. 5b_1_–b_9_) formed by blocks B and C can be observed due to the competition between the stronger incompatibility and the solvophobicity difference.

**Fig. 6:**
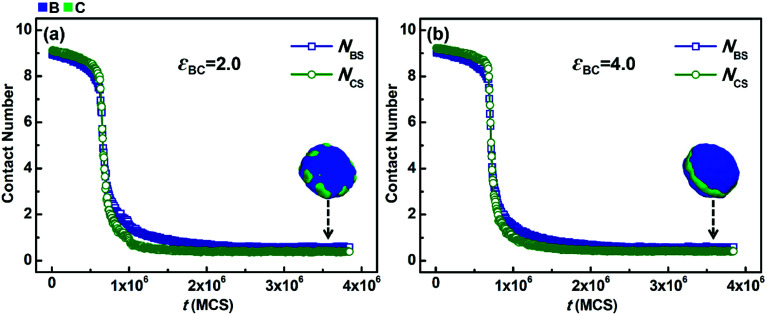
Variations in the average contact numbers *N*_BS_ and *N*_CS_ with simulation time (*t*) in the case of *k*_BC_ = *ε*_BS_/*ε*_CS_ = 1.5 (in which *ε*_BS_ = 6.0 and *ε*_CS_ = 4.0). (a) *ε*_BC_ = 2.0 and (b) *ε*_BC_ = 4.0. For clarity, the morphologies of the solvophobic parts of the corresponding micelles are also given in this figure.

In addition to *N*_BC_, the average contact numbers between the solvophobic blocks and solvents (*N*_BS_ and *N*_CS_), which reflects the phase separation degree between the solvophobic blocks and solvents, were also calculated. Fig. 6 shows the variations in *N*_BS_ and *N*_CS_ with simulation time (*t*) when the solvophobicity difference between blocks B and C is small, *i.e.*, *k*_BC_ = *ε*_BS_/*ε*_CS_ = 6.0/4.0 = 1.5. The situations with weak (*ε*_BC_ = 2.0) and strong (*ε*_BC_ = 4.0) incompatibility between blocks B and C are shown in Fig. 6a and b, respectively. As shown in Fig. 6a, *N*_BS_ and *N*_CS_ rapidly decrease and then remain almost unchanged with an increase in *t*. Both the decreasing speed and equilibrium value of *N*_BS_ are quite similar to that of *N*_CS_. This means that the phase separation speeds of blocks B and C from solvents are close, and the contact area of blocks B with solvents is similar to that of blocks C in the final micelles. This phenomenon is quite reasonable because the solvophobicity difference between blocks B and C is small. A similar phenomenon is also observed in Fig. 6b. This indicates that when the solvophobicity difference is small, increasing the incompatibility between blocks B and C has almost no impact on either the phase separation speeds or the final contact areas between the solvophobic blocks and solvents.

**Fig. 7:**
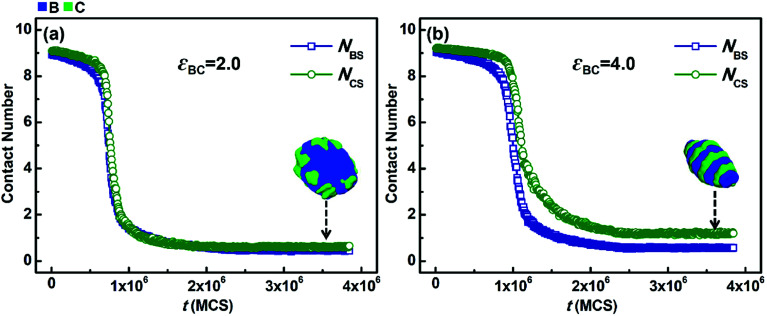
Variations in the average contact numbers *N*_BS_ and *N*_CS_ with simulation time (*t*) in the case of *k*_BC_ = *ε*_BS_/*ε*_CS_ = 2.5 (in which *ε*_BS_ = 5.0 and *ε*_CS_ = 2.0). (a) *ε*_BC_ = 2.0 and (b) *ε*_BC_ = 4.0. For clarity, the morphologies of the solvophobic parts of the corresponding micelles are also given in this figure.

However, when the solvophobicity difference between blocks B and C is large, the situation is different. Fig. 7 shows the variations in *N*_BS_ and *N*_CS_ with *t* when the solvophobicity difference between blocks B and C is relatively large, *i.e.*, *k*_BC_ = *ε*_BS_/*ε*_CS_ = 5.0/2.0 = 2.5. As shown in Fig. 7a, in the case of weak incompatibility (*ε*_BC_ = 2.0), the variation curves of *N*_BS_ and *N*_CS_ almost overlap with each other, which indicates that although the solvophobicity difference between blocks B and C increases, the phase separations of blocks B and C with solvents are still quite similar. However, as shown in Fig. 7b, when the incompatibility between blocks B and C is increased (*ε*_BC_ = 4.0), the decreasing speed of *N*_BS_ is apparently faster than that of *N*_CS_, and the equilibrium value of *N*_BS_ is much smaller than that of *N*_CS_. By comparing Fig. 7b with 7a, it can be found that an increase in the incompatibility between blocks B and C enlarges the effect of the solvophobicity difference between blocks B and C, which results in great changes in the micelle morphology.

From the simulation results shown in Fig. 6 and 7, it can be found that the competition between the incompatibility and solvophobicity difference between blocks B and C governs the microphase structures of the solvophobic cores in the micelles. On the other hand, when the solvophobicity difference between blocks B and C is large, an increase in the incompatibility can markedly enlarge the effect of the solvophobicity difference, which can result in big changes in not only the microstructures of the solvophobic cores, but also the overall micelle morphologies formed by ABC triblock copolymers.

## 4. Conclusions

In this study, the microphase separation behaviors of linear ABC triblock copolymers in A-selective solvents were investigated using Monte Carlo simulation. We mainly focused on the effects of the solvophobicity difference and the incompatibility between solvophobic blocks B and C on the micelle morphologies formed by linear ABC triblock copolymers. Phase diagrams as a function of the solvophobicity of blocks B and C were obtained at different repulsions between blocks B and C, respectively. A series of multicompartment micelles with distinct solvophobic parts were obtained, such as pupa-like multi-layered micelles, hamburger-like micelles and bumpy disks. Notably, when the solvophobicity of blocks B is much stronger than that of blocks C, a novel reverse core–shell–corona micelle with solvophilic blocks A located in the center of the micelle was obtained. By investigating the microphase structures of the solvophobic parts in the multicompartment micelles, it was found that the competition between the effects of the repulsions and the solvophobicity difference between blocks B and C determines the microphase separation structures in multicompartment micelles. Moreover, the simulation results also indicate that an increase in the incompatibility between blocks B and C enlarges the effect of the solvophobicity difference, which results in changes in not only the microstructures of the solvophobic cores, but also the overall micelle morphologies. These simulation results elucidate the mechanism of the formation of ABC triblock copolymer nanostructures and provide a theoretical basis for the precise control of micelle structures in experiments.

## Conflicts of interest

There are no conflicts to declare.

## Supplementary Material
